# Passing the probe: building the next generation of rheumatologists with independent ultrasound practice in Greater Manchester

**DOI:** 10.1093/rap/rkaf132

**Published:** 2025-11-14

**Authors:** Anastasia-Vasiliki Madenidou, Qasim Akram

**Affiliations:** Centre for Musculoskeletal Research, The University of Manchester, Manchester, UK; Kellgren Centre for Rheumatology, Manchester Royal Infirmary, Manchester University NHS Foundation Trust, Manchester, UK; Rheumatology Department, Stepping Hill Hospital, Stockport NHS Foundation Trust, Stockport, UK

Dear Editor, In 2018, Qasim Akram (Q.A.) published an article in *Rheumatology* describing his experience obtaining a EULAR ultrasound accreditation as a UK rheumatology trainee [1]. In 2025, Anastasia Vasiliki Madenidou (A.-V.M.), under Q.A.’s supervision, also achieved this accreditation [2]. In this article, A.-V.M. and Q.A. highlight A.-V.M.’s ultrasound journey as an example of ‘passing the probe’ to the next generation and discuss ongoing challenges in UK ultrasound training.

Ultrasound is becoming an increasingly valuable adjunct to the management of patients with inflammatory arthritis and beyond. In rheumatology clinical practice, ultrasound scans are typically performed by rheumatologists or healthcare professionals with ultrasound training, and by musculoskeletal radiologists. It is important to note that ultrasound is not a replacement for clinical practice but rather an extension, as ultrasound findings should be interpreted in the context of clinical history and relevant investigations.

In 2017, Q.A. achieved the EULAR independent ultrasound accreditation after undertaking an international ultrasound fellowship in Spain and meeting the requirements [2]. Given the lack of ultrasound training centres in the UK, a fellowship outside the UK was essential to achieve the required independent ultrasound skills, especially in a short space of time during higher specialist training and without the constraints of National Health Service (NHS) provision. He had to seek deanery approval, secure funding and find a suitable training centre/supervisor to host him.

After starting his substantive consultant job at Stepping Hill Hospital (SHH), he set up the Stockport Rheumatology Ultrasound Programme. The structured training programme is integrated for trainees throughout the year and includes a blend of theoretical and practical aspects of ultrasound. Q.A. has dedicated time in his job plan for ultrasound teaching, has developed an ultrasound course in Manchester and has taught on both BSR and EULAR ultrasound courses. Q.A. also secured recognition of the Rheumatology Department at SHH as a EULAR Imaging Training Centre.

A.-V.M. received purely UK-based ultrasound training. She began learning musculoskeletal ultrasound (MSUS) during her ST4 placement at SHH under Q.A.’s supervision. She, alongside other trainees, had dedicated time to scan. When A.-V.M. moved to Salford Royal Hospital as an ST5, she was allowed to take study leave to attend ultrasound clinics under Q.A.’s supervision and further enhance her scanning skills. The training under Q.A. continued further during her out-of-programme time when she managed to attend the required face-to-face EULAR ultrasound courses and, finally, pass the practical exams and receive her independent ultrasound accreditation in June 2025. Following Q.A.’s steps, A.-V.M. is also keen to train more clinicians in ultrasound scanning.

Although A.-V.M.’s experience was streamlined thanks to Q.A.’s teaching and regional support, ultrasound training in the UK is still not straightforward. Ultrasound training is not part of the training curriculum [3], which can make it challenging for trainees to use their study leave or budget for ultrasound activities, as other training needs take priority. As of today, there are ongoing efforts by the Rheumatology Specialist Advisory Committee to include ultrasound training as an option in the curriculum, which will allow trainees to learn ultrasound if interested. There has also been discussion of post certificate of completion of training rheumatology ultrasound fellowships.

There are a lot of excellent courses organized by the BSR and other organizations, but it is not possible to become an independent ultrasound practitioner by attending courses. Ultrasound is a practical skill and takes time and practice to become an independent practitioner as well as understand its relevance in a clinical setting. Mentorship and access to supervised ultrasound scanning are paramount to reach independent practice. Despite the efforts of the BSR ultrasound special interest group, there are still only a handful of rheumatology centres offering ultrasound training. Other barriers could include the lack of dedicated teaching time for trainers in their NHS job plans or the limited access to ultrasound machines for trainees.

Given that treatment decisions are based on ultrasound findings, accreditation is important to ensure that practitioners possess the minimum skills required for independent scanning. In the UK, the accreditation options are restricted to university-based courses that offer more of a general MSUS training compared with the EULAR ultrasound pathway, which is specifically focused on rheumatological ultrasound [2]. A BSR accreditation may be an appropriate next step following the introduction of the BSR ultrasound course.

We should also not underestimate the financial burden associated with ultrasound accreditation. Although A.-V.M. was partially funded to participate in some of the required EULAR activities, she had to use additional personal funds to achieve the EULAR accreditation. Revising the study budget or introducing complementary initiatives, such as BSR funding in addition to the existing education bursary, would be beneficial [4, 5].

In conclusion, A.-V.M.’s accreditation showcases that high-level ultrasound training is possible as part of rheumatology training. As Q.A. has done, independent ultrasound practitioners should likewise take the initiative to train their colleagues. We hope that the examples of Q.A. and A.-V.M., together with the proposed solutions to current training challenges, will inspire the next generation of trainers and trainees and help make ultrasound training more widely available.

## Data Availability

Data are available in the article.

## References

[1] Akram Q. Training issues in ultrasound and the benefits of an International Fellowship. Rheumatology (Oxford) 2018;57:947–8. 28505343 10.1093/rheumatology/kex192

[2] EULAR. EULAR MSUS competency assessment | level 1. https://esor.eular.org/course/view.php? id=94 (19 September 2025, date last accessed).

[3] Joint Royal Colleges of Physicians Training Board. Curriculum for rheumatology training. https://www.thefederation.uk/sites/default/files/Rheumatology%25202022%2520Curriculum%2520FINAL.pdf (19 September 2025, date last accessed).

[4] Health Education England. Health Education England (HEE) Study Leave. An overview of the HEE-wide approach. https://www.hee.nhs.uk/sites/default/files/documents/Health%20Education%20England%20%28HEE%29%20Study%20Leave%20-%20An%20overview%20of%20the%20HEE-wide%20approach.pdf (22 September 2025, date last accessed).

[5] British Society for Rheumatology. Education bursary. https://www.rheumatology.org.uk/events-learning/bursaries/uk-member-education-bursary (19 September 2025, date last accessed).

